# A pitfall of bilateral inferior petrosal sinus sampling in cyclic Cushing’s syndrome

**DOI:** 10.1186/s12902-019-0433-9

**Published:** 2019-10-22

**Authors:** Adriana Albani, Christina M. Berr, Felix Beuschlein, Marcus Treitl, Klaus Hallfeldt, Jürgen Honegger, Günter Schnauder, Martin Reincke

**Affiliations:** 1 Medizinische Klinik und Poliklinik IV, Ludwig Maximilian University München, Ziemssenstr. 1, 80336 Munich, Germany; 2 Klinik für Endokrinologie, Diabetologie und Klinische Ernährung, Universitätsspital Zürich, Zürich, Switzerland; 3 Klinik und Poliklinik für Radiologie, Klinikum der Universität München, LMU München, Munich, Germany; 4 Chirurgische Klinik und Poliklinik–Innenstadt Klinikum der Ludwig-Maximilians-Universität München, Munich, Germany; 5 Department of Neurosurgery, Eberhard Karls University Tubingen, Tubingen, Germany; 6 Department of Internal Medicine, Endocrinology and Diabetology, University of Tubingen, Tubingen, Germany

**Keywords:** ACTH, Cortisol, Hypercortisolism, Cushing disease, Neuroendocrine tumor

## Abstract

**Background:**

Clinical care of patients with cyclic Cushing’s syndrome (CS) is challenging. Classical pitfalls include incorrect subtyping, unnecessary surgical procedures and delayed definite treatment.

**Case presentation:**

A 43-year-old female suffered from a rapidly cycling ectopic CS. She experienced six cycles of severe hypercortisolism within a 2 year period (maximum plasma cortisol 5316 nmol/L, normal range 124.2–662.4 nmol/L; maximum urinary free cortisol 79,469 nmol/24 h, normal range < 414 nmol/24 h) lasting 2–9 weeks. The episodes were associated with pronounced hypokalemia (lowest K^+^ value recorded 2.4 mmol/l) and progressive signs and symptoms of CS. A bilateral inferior petrosal sinus sampling (BIPSS) performed during a trough phase was false positive for pituitary ACTH overproduction resulting in unnecessary transsphenoidal surgery while a second BIPSS performed during an active phase was indicative for ectopic CS. The ^18^F-DOPA PET/CT showed a pancreatic lesion, which was subsequently partially removed. Surprisingly, the histopathology was conclusive for ACTH-positive lymph node metastasis located in the retro-duodenal tissue of an occult neuroendocrine tumor WHO grade II. The primary tumor has not been identified so far and, because of the persistent hypercortisolism, the patient underwent bilateral adrenalectomy. Two years later, ACTH levels started to increase progressively. Percutaneous biopsy of a newly identified suspected lesion in the fifth thoracic vertebra revealed a metastasis with positive staining for ACTH, synaptophysin and chromogranin A. Therapy with carboplatin and etoposide was started and, since then, the patient underwent 12 cycles of chemotherapy.

**Conclusions:**

We report the challenging case of a rapidly cycling CS secondary to ACTH-secreting neuroendocrine intestinal tumor of unknown primary. We highlight the importance of performing diagnostic tests only during the phases of active cortisol secretion and as soon as first symptoms appear to avoid pitfalls.

## Background

Cyclic Cushing’s syndrome (CS) was first described in the late fifty [1, 2]. It is a rare disorder, in which cortisol levels are fluctuating, alternating between periods of hypercortisolism and spontaneous remission. The diagnosis of cyclic CS relies on at least three peaks and two troughs of cortisol production [3]. Case reports from the literature have shown a great variability in the length of each period that can ranges from few hours to several months, sometimes with long disease-free intervals [4–6]. The pathophysiology is not clear, although studies of isolated cases have suggested dopaminergic, serotoninergic and other hypothalamic influences [7–9]. Biochemical episodes of cycling occur in 15–36% of cases and they seem to be very rare in children [10–15]. However, its prevalence is likely underestimated, as the diagnosis requires a careful investigation of cortisol pattern secretion. Patients with cyclic CS present most frequently with pituitary tumors (54%), followed by ectopic (26%) and adrenocortical tumors (11%) [14]. The fluctuations of cortisol levels make the diagnosis of CS extremely challenging and, differentiating between central and ectopic secretion, can incur in several pitfalls. Here we report the unusual case of a 43-year-old female with a severe rapidly cycling hypercortisolism secondary to an ectopic occult adrenocorticotropic hormone (ACTH)-positive neuroendocrine tumor (NET) and review the relevant literature.

## Case presentation

**Fig. 1:**
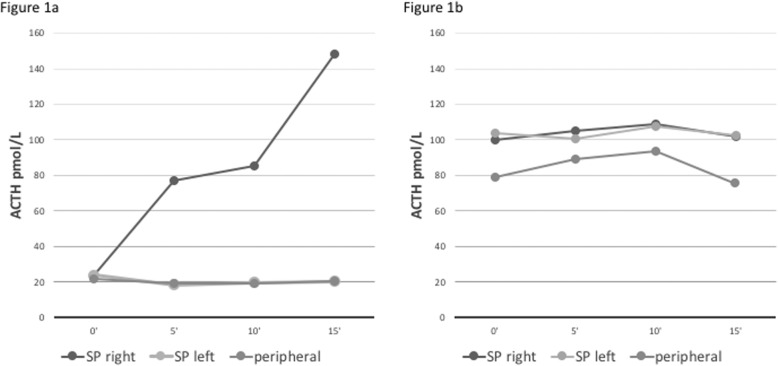
**a** First BIPSS performed during a trough phase wrongly suggestive of Cushing’s disease. **b** Second BIPSS performed during a phase of hypercortisolism suggestive of ectopic ACTH secretion

A 43-year-old female was admitted to a German Hospital in August 2012 for hypokalemia (lowest K^+^ value 2.4 mmol/l) muscle weakness, palpitations and sleeplessness. Patient history and clinical examination showed arterial hypertension controlled by three antihypertensive drugs and peripheral edema. Hyperaldosteronism was excluded, and oral potassium supplementation was started. Symptoms resolved spontaneously after 1 month and the patient did not undergo further examinations. In January 2013 the patient was hospitalized for hypokalemia (lowest K^+^ value 2.6 mmol/l) and uncontrolled arterial hypertension despite three antihypertensive drugs. She complained of weight gain, muscle weakness and oligomenorrhea. Physical examination revealed mild hirsutism and oral candidiasis. Renal arterial stenosis and pheochromocytoma were ruled out. Elevated fasting blood glucose, elevated ACTH (74 pmol/L with normal values 1.98–11.4 pmol/L) and cortisol levels (1648 nmol/L) were documented. A suppression test with 2 mg dexamethasone showed lack of cortisol suppression. Serum cortisol after 8 mg dexamethasone was 513 nmol/L (normal: < 50). An ACTH-dependent CS was suggested. Two months later signs and symptoms of hypercortisolism disappeared, and biochemical remission was documented (Table 1). Pituitary MRI did not identify a pituitary adenoma, while a bilateral inferior petrosal sinus sampling (BIPSS) performed in the off-phase was indicative of central ACTH production because of a strong ACTH increase after corticotrophin release hormone (CRH) injection (100 μg i.v.) in the right petrosal sinus and a central to peripheral gradient of 7.3 at 15′ (Fig. 1a). The patient remained without symptoms for around 5 months. During this time, arterial blood pressure was medically controlled and potassium levels were normal without supplementation. In August 2013 a new episode occurred. The worsening of glucose metabolism required insulin therapy. Basal serum cortisol and 24 h urinary free cortisol (UFC) levels were highly abnormal. A suppression test with 8 mg dexamethasone confirmed lack of cortisol suppression and a new pituitary MRI identified a suspected pituitary lesion. Only 4 weeks later Cushing’s symptoms disappeared, and diagnosis of cyclic CS was established. A 68Ga-DOTATATE PET/CT was negative. The patient underwent explorative transsphenoidal surgery that showed no pituitary adenoma but a Crook’s cell hyalinosis in the pituitary gland. In January 2014, after 4 months, hypercortisolism recurred. A systematic selective venous sampling of all major veins did not identify an ACTH gradient. A CRH stimulation test showed no increase in ACTH and cortisol levels, suspicious for ectopic CS. Four weeks later symptoms disappeared. An 18-FDG-PET CT was negative and the patient was referred to our University Hospital. During this symptom-free interval, midnight salivary cortisol, 24hUFC and 1 mg dexamethasone suppression test remained slightly abnormal (midnight salivary cortisol 121 nmol/L (normal range: < 41); 24hUFC 1051 nmol/24 h; serum cortisol after 1 mg dexamethasone 215 nmol/L (normal range: < 50). In May 2014, the fifth episode occurred lasting for 2 weeks (Table 1). CRH levels were low (8.4 pg/ml), excluding an ectopic CRH secretion. Another BIPSS was performed and was now in line with ectopic CS (Fig. 1b). Abdomen MRI and CT, angio MRI and 68Ga-DOTATATE PET/CT were all negative. An 18F-DOPA PET/CT identified a lesion close to the pancreatic head, which was subsequently confirmed by an endoscopic ultrasound, showing a 13x12mm hypoechoic lesion in the pancreatic head. Subcutaneous pasireotide was administered for 4 days with immediate normalization of cortisol levels, which remained normal thereafter suggesting a spontaneous remission rather than a therapeutic effect. In August 2014, the patient was again symptomatic. Daily salivary cortisol monitoring documented the rapid and steep increase in cortisol concentrations, which reached peak levels in 5 days. The patient was treated with intravenous continuous etomidate to control life-threatening cortisol levels. After discussion in a multidisciplinary tumor board a Whipple’s intervention with extended lymphadenectomy was performed. Surprisingly, histopathology did not confirm the pancreatic lesion but identified several ACTH-positive lymph node metastases. The Ki67 staining was limited due to the strong fragmentation of the tissue and the presence of numerous intratumoral lymphocytes. On average, Ki67-positive tumor cell nuclei were less than 20%. Only in single hot spot region, areas with up to 25 to 30% positive tumor cell nuclei were identified. Together with the clinical data (no further primary suspect focus detectable in the DOPA and DOTATATE PET/CT and MRI), the findings were suggestive for an occult, possibly pancreatic, NET WHO grade II with accompanying loco-region lymph node metastasis, located in the retro-duodenal tissue close to the pancreatic head. The tumor infiltrates reached the broken surface of the specimen, indicating an incomplete tumor resection. Post-surgical ACTH levels dropped from 182 to 82 pmol/L and remained stable between 44 and 66 pmol/L for around 1 month. Because of the incomplete tumor resection, the patient underwent bilateral adrenalectomy, resolving symptomatology. The primary tumor remained occult and, 2 years after adrenalectomy, ACTH levels started to increase progressively, reaching values of 2676 pmol/L in January 2018. At the same time, chromogranin A raised to 1586 μg/L, compared with 418 μg/L in July 2017. Dopa PET/CT and MRI of the spine, performed in November 2017 and January 2018 respectively, showed multiple sclerotic lesions suggestive of bone metastases. A suspected lesion of the fifth thoracic vertebra underwent percutaneous transpedicular biopsy. Pathology report documented an ACTH-, synaptophysin- and chromogranin A-positive metastasis of the NET tumor with a proliferation rate of 80%. After discussion in multidisciplinary tumor board, the patient started therapy with carboplatin and etoposide and, since then, has received 12 cycles of chemotherapy. Imaging studies documented unchanged number and size of target lesions, reflecting stable disease. Chromogranin A levels dropped down to 554 μg/l in February 2018, but reached again a peak of 2657 in June 2018. The last value, in January 2019, is 1213 μg/L. ACTH levels continued to increase over the time, reaching in October 2018 values of 11,257 pmol/L.

**Table 1:** Biochemical data at the time of the first and the second BIPSS

	Values	Normal values
Biochemical data at the time of the first BIPSS		
ACTH (pmo/L)	14	< 11
Basal cortisol (nmol/L)	566	< 630
UFC nmol/24 h	434	< 789
Biochemical data at the time of the second BIPSS		
ACTH (pmo/L)	120	< 11
Basal cortisol (nmol/L)	4858	< 662
UFC nmol/24 h	67,857	< 414

## Discussion and conclusions

**Fig. 2:**
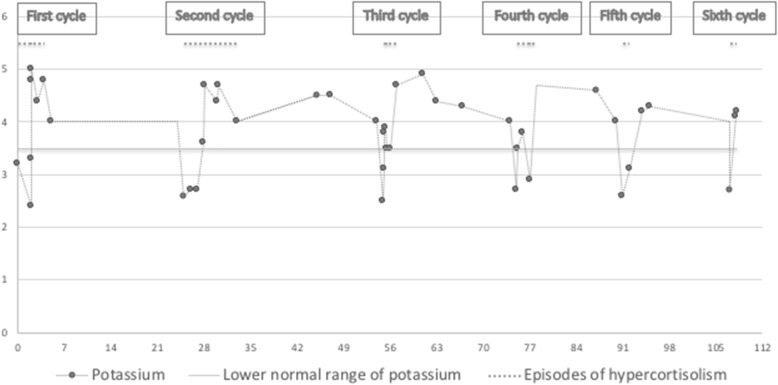
Potassium levels over the time (weeks). Oral potassium supplementation was regularly started after diagnosis of hypokalemia

Diagnosis and subtyping of cyclic CS are difficult, especially when periods of hypercortisolism are short and interspersed by periods of near-normal cortisol levels. Several pitfalls are possible, and we report here such a classical scenario in a patient with rapidly cycling hypercortisolism. The patient experienced, within a 2-year period, six cycles lasting usually not more than 4 weeks, with pronounced hypokalemia occurring in each phase (Fig. 2). In such a situation, results of biochemical testing might be conflicting, and therapeutic decision making will often be prolonged. In our patient, identification of the ACTH source turned out to be extremely challenging. The BIPSS performed during a phase of normal cortisol secretion raised the suspicion of Cushing’s disease (Fig. 1a) and lead to unsuccessful pituitary surgery, also because initial 68Ga-DOTATATE PET/CT imaging did not reveal an ectopic source. The second BIPSS during an ‘on phase’ of CS lead to the correct diagnosis, showing a typical ectopic pattern without any gradient (Fig. 1b). In a similar case, BIPSS performed during a trough phase was misleading, wrongly suggesting a peripheral ACTH source [16]. The confirmation of high basal serum cortisol on the day of performing BIPSS could be helpful to avoid diagnostic failure and unsuitable testing [17]. Other mistakes in the diagnostic workout of cyclic CS might be due to discrepancies of test results. For example, cortisol response to dexamethasone suppression test can be misleading, reflecting increased or decreased levels of cortisol secretion [3, 18]. The real prevalence of cyclic CS is hard to establish because of many difficulties related to the diagnosis. In a review of 65 patients with cyclic CS, pituitary tumors accounted for 54%, followed by ectopic (26%) and adrenocortical tumors (11%). Considering the low prevalence of patients with CS secondary to ectopic tumors, an ectopic source of ACTH hypersecretion seems to be more frequent in patients with biochemical episode of cycling [14]. Neuroendocrine tumors of the thymus are the most frequently represented, followed by neuroendocrine bronchial tumors [4, 19–26]. Other tumors reported as responsible of cyclic ectopic CS are pancreatic, renal and gastric NET [27–29], epithelial thymoma [30], phaeochromocytoma [31], carotid glomus tumor [32] and ectopic pituitary adenoma [33]. Surprisingly, although small cell lung carcinoma is frequent in patients with ectopic CS, biochemical episodes of cycling seem to be rare. Differentiating between ectopic and central ACTH source in cyclic CS is extremely challenging and the localization of the tumor can be very difficult, especially when it is not visible by imaging, as the fluctuations of cortisol levels may lead to several pitfalls. In around 13% of cases, the primary tumor remains unknown [14]. The 68Ga-DOTATATE PET/CT is the first-line PET-imaging and seems to have more accuracy compared with 18F-DOPA-PET/CT [34]. However, in our patient 18F-DOPA PET/CT was suggestive for a pancreatic lesion, which was not identified by 68Ga-DOTATATE PET/CT. Another case, in which 18F-DOPA-PET/CT but no 68Ga-DOTATATE PET/CT was able to identify the tumor lesion, has been reported [35]. In our patient, the histopathology was unexpected, identifying the ‘pancreatic’ lesion as lymph node metastasis of an occult NET located in the retro-duodenal tissue adjacent to the pancreatic head. Because of the incomplete tumor resection, the patient underwent bilateral adrenalectomy, resolving the symptomatology. Bilateral adrenalectomy is a safe and effective treatment when the primary source of ACTH secretion is not surgically removable [36]. An Italian multicentre study conducted in patients with ectopic CS showed a better survival rate in those who underwent adrenalectomy, in the first 2 years after surgery [37]. Several factors may affect the prognosis in ectopic CS, such as severity of hypercortisolism with relative comorbidities, presence of hypokalaemia, type and grade of the NET and presence of metastases [37]. Avoiding all the potential pitfalls is of primary importance in order to speed-up the diagnosis and prevent unnecessary treatments. In summary, we reported here the challenging case of a patient with a rapidly cycling Cushing’s syndrome secondary to ACTH-secreting neuroendocrine intestinal tumor of unknown primary, in whom a BIPSS performed during a trough phase was wrongly suggestive of central ACTH overproduction. This case highlights the potential pitfalls occurring in diagnosis, subtyping and localization of a tumor, which is cyclically the source of ACTH hypersecretion. It also demonstrates the importance of performing diagnostic tests only during the phases of active cortisol secretion, as soon as first symptoms appear. In addition, our case is peculiar because of the histopathology of the suspected pancreatic lesion, surprisingly conclusive for lymphonode metastasis of an occult NET located in the retro-duodenal tissue. To our knowledge, no similar cases have been reported in the literature.

## Data Availability

Not applicable.

## Abbreviations

ACTH: Adrenocorticotropic hormone
BIPSS: Bilateral inferior petrosal sinus sampling
CRH: Corticotrophin release hormone
CS: Cushing’s syndrome
NET: Neuroendocrine tumor
UFC: Urinary free cortisol
